# Editorial: Organ cross talk and its impact on the clinical course in multiple trauma and critical illness

**DOI:** 10.3389/fimmu.2023.1195371

**Published:** 2023-04-05

**Authors:** Klemens Horst, Tom Eirik Mollnes, Markus Huber-Lang, Frank Hildebrand

**Affiliations:** ^1^ Department of Orthopaedics, Traumatology and Reconstructive Surgery, Rheinisch-Westfälische Technische Hochschule (RWTH) Aachen University, Aachen, Germany; ^2^ Department of Immunology, Oslo University Hospital and University of Oslo, Oslo, Norway; ^3^ Research Laboratory, Nordland Hospital Trust, Bodo, Norway; ^4^ Institute of Clinical and Experimental Trauma-Immunology, University Hospital of Ulm, Ulm, Germany

**Keywords:** organ cross talk, multiple trauma, polytrauma, critical ill, clinical course

Communication between different body compartments that can also affect remote tissues has gained increasing interest. This so-called “organ-cross-talk” seems to be of major relevance after multiple trauma (MT) and critical illness (CI). In this context, systemic effects of injuries to different body compartments have been shown to be far greater than the sum of the isolated traumatic insults and posttraumatic dysfunction of primarily unaffected organs. This underscores the importance of a holistic research approach when studying the complex biology and pathophysiology in this field.

Posttraumatic and/or post-surgical inflammation have a substantial impact on patient outcome. Maleitzke et al. presented rare data on clinically relevant laboratory parameters in patients suffering from penetrating trauma. Aside from the injury severity score (ISS), the authors found that AST, CRP, erythrocyte count, pH, lactate, aPTT and K+ were useful for identifying patients at risk and adjusting surgical and ICU algorithms early on. Both detrimental and regenerative roles of inflammatory mediators have been described in numerous pre-clinical studies and experimental settings. For example, Ragipoglu et al. investigated the role of mast cells that have the potential to trigger local and systemic inflammation in bone repair in a murine model. The authors found that combined trauma resulted in compromised bone repair in mast-cell component mice. It was concluded that mast cells could represent a potential target for new treatment options to improve fracture healing in patients with multiple trauma. Another murine model, presented by Mkrtchian et al., investigated the role of extracellular vesicles (EVs) as potential mediators transferring information from the injured tissue to remote organs, including the brain. The authors concluded that surgical procedures alter the cargo of circulating EVs in the blood, thus regulating metabolic processes. Zhang et al. reported on systemic inflammatory consequences and focused on traumatic brain injury (TBI). As the brain and the spleen are connected *via* autonomic innervation and by soluble mediators, the authors hypothesized that ethanol intoxication (EI), the most common comorbidity of TBI, influences the peripheral inflammatory response. They concluded that TBI induces rapid maturation of immunomodulatory functions of dendritic cells that is enhanced by EI prior to TBI.

Lackner et al. demonstrated cardiac alterations after hip fracture with enhanced myocardial expression of HMGB1, TLR2/4, TNF, IL1-β and NLRP3, as well as considerable alterations in the myocardial expression of glucose and fatty acid transporters (HFABP, GLUT4) in middle-aged (52-week-old) mice. Hemorrhagic shock (HS) and hypoperfusion influence the immunologic reaction in MT. As lung co-morbidities can aggravate tissue hypoxia *via* alveolar hypoxia, Wepler et al. hypothesized that glucocorticoid receptor (GR) function in mice and pre-traumatic cigarette smoke (CS) exposure would further impair hemodynamic stability and organ function after HS. As the authors were not able to explain whether the observed metabolic acidosis or increased lipolysis was responsible for the trend towards lower catecholamine requirements in CS-exposed mice, further studies are warranted. Halvachizadeh et al. studied the role of occult hypoperfusion (OH), defined by persistent lactic acidosis despite normalization of vital parameters. In a porcine model, the authors found that OH is associated with decreased local circulation and increased local inflammation in the injured soft tissue of the extremity in polytrauma, thus probably reflecting the severity of local soft tissue injuries and potentially guiding treatment strategies.

Following extensive trauma surgery, Teuben et al. recorded a shift in composition of the bone marrow (BM) neutrophil pool, which was associated with relative circulatory neutropenia in a porcine polytrauma model. Moreover, the authors reported that the CXCR4^high^-neutrophil subset became overrepresented, possibly reflecting remigration of aged neutrophils into the BM. These findings may contribute to the development of novel interventions aimed at modifying the trauma-induced response in BM. Air embolism is a serious and underdiagnosed complication during invasive medical procedures. Storm et al. investigated the role of air embolism in a porcine model of thromboembolism. The authors described activation of complement mainly at the level of C3, with subsequent cytokine release, and concluded that C3-inhibition might represent a therapeutic approach to attenuate this response.

A feared complication in MT remains the development of sepsis and multi organ dysfunction syndrome (MODS). Nakanishi et al. focused on the prolonged physical dysfunction in MT patients and investigated the role of neutrophils in muscle atrophy in murine sepsis. They found that sepsis is causal for infiltration of neutrophils in muscles, leading to muscle atrophy and weakness. Of note, these findings were reversed by neutrophil depletion. Gray et al. also reported data from a murine sepsis model. The authors investigated the V-domain Immunoglobulin Suppressor of T cell Activation (VISTA) as it is a potential candidate for strategic targeting in sepsis. The group proposed a protective Treg-mediated role for VISTA by which inflammation-induced tissue injury is suppressed and improves survival in early-stage sepsis. The authors concluded that enhancing VISTA expression or adoptively transferring VISTA+ Tregs in early-stage sepsis may represent a novel therapeutic approach to ameliorate inflammation-induced death. Patel et al. focused on macrophage migration inhibitory factor (MIF) in MT patients and a rat model of HS and the potential of MIF inhibitor ISO-1 to reduce MODS in this model. The authors described increased MIF levels in MT patients and HS rats. Whereas MIF caused organ injury and/or dysfunction and hypotension in rats, treatment with ISO-1 attenuated organ injury and dysfunction, and reduced the activation of NF-kB and NLRP3 pathways in the rat kidney and liver. Thus, the authors pointed out that MIF inhibitors may be used as a potential therapeutic approach for MODS after trauma and/or hemorrhage. Sulforaphane (SFN) was also reported to exert beneficial immunomodulatory effects in a murine model of HS and resuscitation as described by Liang et al. They proved that *in vivo* SFN treatment can decrease HS/resuscitation-induced hepatic ischemia-reperfusion injury and modulate the activity of Kupffer cells *via* an Nrf2-dependent pathway. To better understand the role of complement component 1 inhibitor (C1-INH), Nielsen et al. established a novel porcine model of ischemia-reperfusion injury (IRI) by cross-clamping the thoracic aorta. The authors evaluated the global changes occurring in organ function, systemic inflammatory response and organ damage with or without treatment with C1-INH-concentrate. Although C1-INH treatment did not have any significant effects, probably due to its low specificity and efficacy in inhibiting complement, the model itself was found to be valid for future testing of drugs to combat IRI.

Messerer et al. conducted a study to investigate possible drug effects in a porcine model of HS. The group investigated the role of the H2S donor sodium thiosulfate (Na_2_S_2_O_3_, STS). As the authors had previously found beneficial effects in mice and pigs with co-morbidities such as coronary artery disease, they conducted a prospective, randomized, controlled, blinded experimental study to address the effects of STS in cardiovascular healthy pigs. However, Na_2_S_2_O_3_ did not show any benefits in healthy organisms undergoing HS. Lupu et al. focused on complement activation and Toll-like-receptor signaling immediately after trauma. They assessed the efficacy of the combined inhibition therapy of complement factor C5 and TLR co-receptor CD14 on thrombo-inflammation and organ damage in a 72-h porcine model of MT and found that combined C5 and CD14 inhibition limited the catecholamine demand, the inflammatory response, and signs of organ damage after experimental polytrauma, which might indicate a promising therapeutic approach. Shah et al. focused on multi-system inflammation and organ dysfunction after trauma and HS in a murine model. By hypergraph analysis and principal component analysis of 20 proteins sampled from the heart, gut, lung, liver, spleen, kidney and systemic circulation, the authors found that IL-17A was present persistently in all tissues at all sampled time points (except for its absence in the plasma at 0.5 h) in the wild type strain compared to TLR4-null (TLR4 -/-) animals. The authors concluded that trauma and HS induced the efflux of Th17 cells from the circulation and into specific tissues, suggesting a complex, content-specific role for TLR4 and type 17 immunity following trauma and HS.

In conclusion, this Research Topic presents the latest scientific insights on immunological and cellular reactions among different organs after severe trauma or during the further clinical course of critically ill patients. The data presented will deepen our understanding of the underlying mechanisms in regard to organ-cross-talk as well as potentially improving the treatment of patients post trauma or during intensive care. We thank all authors for their contribution.

## Author contributions

KH, TM, MH-L, and FH defined the topic of this Research Topic. All authors contributed to the article and approved the submitted version.

